# The relationship between health literacy and satisfaction with medical care in parents of children with chronic gastrointestinal diseases

**DOI:** 10.3389/fpubh.2026.1828560

**Published:** 2026-04-29

**Authors:** Agnieszka Pacut, Mariusz Duplaga, Sabina Więcek, Izabela Murzyn, Gabriela Prusik, Elzbieta Krzesiek, Natalia Stalony, Kinga Kowalska-Duplaga

**Affiliations:** 1 Doctoral School of Medical and Health Sciences, Jagiellonian University Medical College, Krakow, Poland; 2 Department of Health Promotion and e-Health, Institute of Public Health, Faculty of Health Sciences, Jagiellonian University Medical College, Krakow, Poland; 3 Department of Pediatrics, Medical University of Silesia, Katowice, Poland; 4 Department of Pediatrics, Gastroenterology, Hepatology, Nutrition, Allergology and Pulmonology, Medical University of Bialystok, Bialystok, Poland; 5 Department of Pediatrics, Gastroenterology and Nutrition, Wroclaw Medical University, Wroclaw, Poland; 6 Department of Pediatric Gastroenterology and Nutrition, Medical University of Warsaw, Warsaw, Poland; 7 Department of Pediatrics, Gastroenterology and Nutrition, Medical Faculty, Jagiellonian University Medical College, Krakow, Poland

**Keywords:** e-health literacy, gastrointestinal disease, health literacy, pediatric chronic disease, satisfaction with medical care, stress

## Abstract

**Introduction:**

Parents' satisfaction with pediatric care is often measured for single encounters, although families managing chronic conditions accumulate experiences across multiple contacts and settings. We aimed to identify predictors of parents' cumulative satisfaction with prior pediatric care, focusing on health literacy, e–health literacy, Internet and social media use, and perceived stress, while accounting for sociodemographic and illness-related factors.

**Methods:**

A cross-sectional paper-and-pencil survey was administered to parents of children treated for chronic gastrointestinal diseases in five university gastroenterology centers in Poland. Parental satisfaction was measured with a study-specific, *ad hoc* 10-item Parental Cumulative Satisfaction with Pediatric Care Scale. Predictors were examined using hierarchical linear regression with four blocks: sociodemographic variables, illness and service-use indicators, perceived stress, and health literacy, e-health literacy, and media-use indicators.

**Results:**

Participants were predominantly mothers (82.4%), mean age was 42.0 years (SD = 5.8), and 66.0% cared for a child with inflammatory bowel disease. Mean satisfaction was high (4.9, SD = 0.81). In regression, explained variance increased from *R*^2^ = 0.094 (sociodemographic block) to *R*^2^ = 0.106 (adding illness/service-use), *R*^2^ = 0.154 (adding stress), and *R*^2^ = 0.202 in the full model. In the full model, higher stress predicted lower satisfaction (B = −0.03; 95%CI−0.04 to−0.02), and inadequate HL (vs sufficient) was associated with lower satisfaction (B = −0.64; 95%CI−0.98 to−0.31). Additional independent correlates included low income ≤ 1,500 PLN (B = −0.37; 95%CI−0.61 to−0.12), bachelor's education (B = 0.40; 95%CI 0.09–0.71), being married/partnered (B = −0.34; 95%CI−0.59-−0.10), and residence in a city >500,000 (B = −0.25; 95%CI−0.47-−0.04). e-Health literacy and media-use indicators were not independently associated.

**Conclusions:**

Cumulative parental satisfaction was significantly associated with the level of perceived stress and health literacy, whereas illness and service-use markers and broad indicators of digital engagement showed limited incremental value after adjustment.

## 1 Introduction

Childhood chronic conditions are commonly conceptualized as health problems that persist over time, require ongoing clinical management, and can limit everyday functioning, participation, or development (1). Despite the epidemiologic and clinical heterogeneity of chronic pediatric illness, a shared feature is the reconfiguration of family life around sustained caregiving work. Parents must involve themselves in symptom surveillance, medication routines, dietary or behavioral regimens, and the logistics of monitoring and follow-up into daily routines, often while balancing employment and care for other family members. Across conditions, caregiving extends beyond practical tasks to continuous management of uncertainties, such as exacerbations and ambiguous symptoms. Parents are also expected to make repeated judgments about when professional support is needed. They must play a role of everyday clinical decision makers translating medical recommendations into concrete actions in the home environment (2). In a consequence, parents of children with chronic illness experience poorer health and psychological outcomes compared with parents of healthy children (3). A systematic review evidenced worse parent health outcomes across multiple domains, including mental health indicators (3).

Elevation of parenting stress among parents of children with chronic conditions is caused by many stressors, including treatment demands, health service use, and illness-related disruptions (4). The persistence of stress over time is also important. A meta-analysis by Pinquart (2018) further supported higher parenting stress among caregivers of children with chronic physical conditions, underscoring the strength and consistency of this effect across diagnoses and settings (5). These findings show that chronic pediatric illness is not solely a medical trajectory for the child but a sustained challenge for the family, shaped by the interaction between clinical burden, care organization, and the resources available to parents.

The caregiving burden depends not only on the clinical severity of the child's condition but also on how care is organized and delivered. Frequent outpatient appointments, diagnostic tests, and hospitalizations increase time pressure and add practical tasks, such as paperwork, scheduling, and coordinating across providers. When care is distributed across multiple settings and specialists, parents often become the main source of continuity, carrying information between visits and helping different teams stay aligned (6). In such arrangements, communication and care coordination are main determinants of how families experience the health system, especially when services span several sectors (6).

Care for a child with a chronic condition requires parents to take on ongoing responsibilities that extend beyond day-to-day caregiving. They commonly manage appointments and referrals, support adherence to treatment plans, monitor symptoms, and advocate for the child's needs during clinical decision-making (2). In this context, a cumulative assessment of care reflects more than general attitudes toward services. It captures whether interactions with the health system are workable over time and whether families receive coordinated support that matches the child's needs.

Chronic gastrointestinal diseases illustrate these issues with particular clarity. Pediatric inflammatory bowel disease (IBD), for example, often involves fluctuating symptoms, substantial self-management demands, and complex medication regimens, with recurrent monitoring and treatment adjustments. Qualitative work on pediatric IBD has identified barriers experienced by patients, parents, and providers, including access-related obstacles, information gaps, and coordination challenges, emphasizing that care barriers are multifactorial and occur across the clinical pathway (7).

In such conditions, parents' evaluations are shaped by repeated interactions over long periods marked with exacerbations, transitions between services, and ongoing efforts to implement physicians' advice in everyday life. This cumulative perspective is especially relevant when the outcome of interest is overall satisfaction with pediatric care based on prior experiences rather than satisfaction with a single encounter.

Satisfaction with medical care has long been treated as a key domain of health care quality, complementing indicators of effectiveness and safety by capturing recipients' evaluations of interpersonal care, communication, access, and responsiveness (7). Traditional frameworks define patient satisfaction as the extent to which care meets an individual's needs and expectations, highlighting its subjective and multidimensional nature (8). Because satisfaction is shaped by expectations and values, it differs conceptually from patient experience, which is typically operationalized as reports of whether specific processes occurred (e.g., understandable explanations, respectful communication) (8). Contemporary measurement literature cautions against treating “experience” and “satisfaction” as interchangeable constructs and emphasizes that they capture related but distinct aspects of care evaluation (9).

In pediatrics, measurement and interpretation are further complicated by the frequent use of parent-proxy reports. Children's direct evaluations may differ from parental assessments because children attend to different facets of care and have different expectations, whereas parents often evaluate both clinical interactions and the practical feasibility of implementing recommendations in daily life. In settings such as pediatric surgery, systematic review evidence shows variation in how child- and family-reported measures define and operationalize experience and satisfaction, emphasizing the need for conceptual clarity when selecting outcomes and interpreting findings (10).

Despite these complexities, satisfaction remains an important outcome because it reflects whether care has met families' needs and expectations and whether services are usable in everyday caregiving. Although satisfaction and experience are conceptually distinct, satisfaction judgments are typically formed on the basis of accumulated care experiences; therefore, evidence derived from patient-experience measures is directly relevant for interpreting satisfaction as an outcome (9). Reviews of patient experience surveys indicate that more positive reported experiences are associated with better adherence to recommended care processes, improved clinical outcomes, better safety, and lower health care utilization. This pattern suggests that patient-centered evaluations indicate meaningful aspects of care quality and are linked to outcomes, rather than reflecting only respondent's impressions (11, 12). In pediatric chronic disease contexts, these links may be even stronger because adherence, follow-up, and day-to-day management depend heavily on caregiver understanding and engagement. Communication quality is repeatedly identified as central to engagement and self-management, particularly when regimens are demanding (13). For families managing chronic illness, satisfaction may therefore function both as an evaluative outcome and as a marker of whether care processes adequately support sustained caregiving work.

Much of the satisfaction literature addresses mainly discrete episodes of care (e.g., one hospitalization, one outpatient visit), which is understandable for service-level quality improvement, and is reflected in commonly used encounter-bounded instruments such as standardized inpatient surveys (14). However, for chronic conditions, parents usually form their overall judgment from repeated contacts with the healthcare system, not from a single visit. Their view often reflects whether they can see the same clinicians over time, whether different professionals give consistent information, how quickly they can access help when needed, and whether the advice they receive is practical to use at home. This longer view matters because families move through recurring phases of stability, monitoring flare-ups, and recovery, with changing needs for services and clear information. For this reason, outcomes reported by patients and families should be read as signals of how well care is connected and coordinated across time, and how well the health system's demands match what the family can realistically manage, rather than as a verdict on isolated encounters (6, 15).

In family-centered care, pediatric quality is explicitly linked to partnership with families, respect for family preferences, shared decision-making, and support for parents' competence in caregiving roles (16). A systematic review focusing on children with special healthcare needs found evidence that family-centered care is positively associated with outcomes that include satisfaction, access, communication, and family functioning (17).

In chronic pediatric illness, the positive associations reported between family-centered care and parental outcomes, particularly satisfaction, perceived communication quality, and perceived access, are understandable because parents must repeatedly translate clinical recommendations into home routines and coordinate care across encounters and providers (16, 17). When services provide consistent, practical guidance, support continuity, and actively involve parents in care planning and decision-making, families are less likely to experience care as fragmented or unpredictable, conditions that can otherwise sustain uncertainty and affect trust (6, 15). In turn, greater perceived support and coherence of care can translate into more favorable global evaluations of pediatric care (16, 17). From this perspective, satisfaction in chronic pediatric care can be seen as an overall judgment of whether the healthcare system helps parents carry out caregiving tasks effectively and safely, while avoiding unnecessary complexity and preventable sources of stress.

Parents' evaluations of pediatric care unfold within an emotional and cognitive context shaped by chronic stress exposure. The Perceived Stress Scale conceptualizes stress as the degree to which individuals assess situations as unpredictable, uncontrollable, and overloaded (18). Such approach oriented on appraisal aligns with broader stress theories emphasizing that stress arises not only from objective demands but from perceived imbalance between demands and coping resources (19). In chronic pediatric illness, parents encounter repeated stressors: symptom fluctuations, uncertainty about prognosis, high treatment demands, and disruptions to family routines. Previous studies indicate that parenting stress is elevated in this population, with illness-related stress linked to treatment demands and service use (4, 5).

Stress may influence satisfaction through several pathways. First, high stress can make it harder to concentrate and process information during clinical encounters, so explanations may feel less clear or harder to apply at home. Second, stress can heighten worry and reduce tolerance for uncertainty, which may strengthen perceptions of insufficient support when recommendations are complex or when outcomes are not fully predictable (20). Third, stress may lead parents to seek information outside clinical settings, including online searching, which can shape how they interpret clinical advice and communication. In this way, perceived stress can be viewed as a near-term context that affects how parents experience communication, shared decision-making, and care coordination, and how they combine these experiences into an overall evaluation of care.

Health literacy (HL) is widely defined as the knowledge, motivation, and competencies to access, understand, appraise, and apply health information to make decisions across health care, disease prevention, and health promotion (21). This definition highlights HL as a functional capability rather than a narrow reading skill. In chronic pediatric care, parental HL is embedded in everyday tasks such as interpreting symptoms, understanding medication and monitoring instructions, preparing questions for consultations, and translating recommendations into feasible routines at home. Evidence from adult populations indicates that limited HL is associated with poorer health outcomes and greater difficulty with understanding and using medical information (22). Pediatric-focused evidence also supports the relevance of parental HL. A systematic review found that parental HL is linked to children's health behaviors and health outcomes, suggesting that limited parental HL can act as a barrier to effective chronic illness management (23).

HL may influence parental satisfaction with healthcare through several mechanisms. This interpretation is also broadly consistent with evidence from adult primary care linking HL with perceived patient-centered communication, shared decision-making, and satisfaction, although these relationships appear to be modest and context-dependent (24). In pediatric care, limited parental HL has been associated with more perceived barriers to accessing care and with a stronger preference for clinician-led rather than more participatory decision-making (25). Studies in pediatric subspecialty settings further suggest that lower parental HL may hinder comprehension of key information about treatment, including elements of the child's treatment plan and home-care instructions (26, 27). Consistent with this, parents with lower HL were more likely to make medication dosing errors, indicating that routine caregiving tasks often place substantial literacy-related demands on families (28, 29). Rather than reflecting parental skills alone, satisfaction may therefore depend on how well the healthcare system's communication, information, and navigation demands match what families can realistically understand and implement in everyday care (30, 31).

This reasoning is consistent with the concept of a health-literate organization, which emphasizes that health services should actively reduce avoidable complexity by making information easier to understand, simplifying navigation across services, and supporting people in using care appropriately (32). The results of prior studies suggest that higher organizational HL is associated with greater patient satisfaction, indicating that system design can improve experiential outcomes beyond individual-level competencies (33).

Digitalization has reshaped how parents manage childhood chronic illness: many routinely use websites, apps, and social media to interpret symptoms, learn about treatments, and seek peer support, particularly when clinical guidance feels hard to translate into everyday routines (34, 35). In this context, e-health literacy (eHL) refers to the ability to seek, find, understand, and appraise health information from electronic sources and apply it to a health problem (36). Beyond technical skills, it involves judging credibility and risk information and integrating online content with professional advice. Higher eHL has been linked to more favorable shared decision-making experiences and better capability wellbeing in adult care, suggesting potential relevance for parents navigating complex pediatric pathways (37). Digital engagement may shape satisfaction by recalibrating expectations, strengthening or undermining trust when online information aligns or conflicts with clinicians, and compensating for perceived informational gaps; it may also increase worry when parents encounter misleading or emotionally intense content, especially under high stress.

Parents' satisfaction with chronic pediatric care may vary with the child's illness trajectory and the frequency of service use. Variables such as the child's age, duration of illness, diagnosis, and the number of outpatient visits and hospitalizations reflect both clinical burden and the family's repeated contact with the health system. More frequent contact can help parents build relationships with clinicians and support continuity, but it also increases the chances of delays, inconsistent information, and organizational problems. In a Norwegian national survey of inpatient pediatric care, several aspects of care delivery (including unexpected waiting and the number of previous admissions) were associated with multiple parent-reported experience domains, illustrating how repeated encounters and service processes can shape overall evaluations of care (38). Diagnosis-specific studies report similar challenges. For example, in pediatric inflammatory bowel disease, focus groups with patients, parents, and providers identified barriers related to access and care processes, showing that problems can accumulate across repeated encounters and become more visible over time (39).

Service intensity can influence satisfaction through relational (communication, trust, continuity) and logistical factors (accessibility, coordination, administrative burden). Parents of children with complex chronic conditions perceive continuity as being connected over time, especially in relation to informational and management continuity (40). They also describe frustration when care is fragmented across providers and settings (40).

Sociodemographic resources intersect with these processes because they shape what families can access and how demanding the care pathway becomes. For example, in a study of pediatric autism diagnostic experiences, parental satisfaction differed by maternal education and family income, suggesting that families can evaluate services differently even within a broadly similar clinical pathway (41). Education and socioeconomic position are also linked to HL and eHL and can affect confidence in navigating services, advocating for the child, and interpreting risk information. Geographic location and access to specialized centers may add travel burden and weaken continuity, which can be particularly important in specialist chronic care (42, 43). These sociodemographic influences are likely to work largely through more immediate factors, such as literacy-related skills, digital access, and stress exposure, rather than through socioeconomic indicators alone. Taken together, this supports the view that parental satisfaction reflects both health system performance and the family's capacity to translate care into workable everyday routines.

Parental satisfaction with pediatric care in chronic illness reflects more than a single encounter. It is shaped by how care is delivered and organized (communication, continuity, coordination, and family-centered practice), by parental resources for using information (HL and eHL), by psychological strain (perceived stress), by the child's illness course and service intensity, and by parents' digital information seeking. Chronic care frameworks emphasize that long-term care depends on sustained interactions between care teams and informed families (44), while quality frameworks treat patient-reported evaluations as a core element of quality assessment (7). However, it remains unclear whether HL and eHL explain parents' overall satisfaction after accounting for sociodemographic resources, the child's clinical burden, and parental stress, especially in conditions with high information needs (34, 39).

The objective of this study was to identify predictors of parents' overall satisfaction with prior pediatric care using hierarchical linear regression, with sequential blocks covering parent sociodemographic characteristics, child and illness-related variables, perceived stress, and finally HL, eHL, and Internet and social media use. We hypothesized that higher HL would be associated with higher satisfaction and would contribute additional explained variance beyond the earlier blocks. We further hypothesized that eHL would show an independent association with satisfaction after adjustment. Finally, we expected that Internet and social media use would remain associated with satisfaction in the fully adjusted model, reflecting their role in information seeking and expectation setting.

## 2 Material and methods

### 2.1 Survey

Data were collected with a paper-and-pencil questionnaire administered to parents of children treated for chronic gastrointestinal diseases between May and December 2023. The study was carried out in five university gastroenterology centers located in Kraków, Wrocław, Warszawa, Katowice, and Białystok (Poland). Parents were approached during the child's hospitalization or at an outpatient appointment. Eligible participants were parents or legal guardians of patients younger than 18 years who were receiving care at one of the participating centers and provided informed consent. Parents of children whose chronic symptoms had been present for less than 6 months were excluded.

The sample comprised 562 parents recruited using convenience sampling. Assuming a target population of more than 1,000,000 parents caring for children with chronic conditions, a 95% confidence level, and a conservative population proportion of 50%, the corresponding margin of error for this sample size is approximately 4.1%. Even if parents of children with chronic gastrointestinal disorders represent less than one-tenth of the broader population of parents of children with chronic conditions, the sampling error would be expected to remain similar under the same assumptions.

The study was approved by the Bioethics Committee of the Jagiellonian University (Decision No. 1072.6120.244.2019 with amendments). All invited parents received information about the study aims and procedures. Written informed consent was obtained from each participant prior to completion of the questionnaire.

### 2.2 Questionnaire

The questionnaire comprised 84 items. It incorporated a set of validated instruments: the 16-item European Health Literacy Survey Questionnaire (HLS-EU-Q16) (45, 46), the 8-item e-Health Literacy Scale (eHEALS) (47, 48), the 7-item Generalized Anxiety Disorder scale (GAD-7) (49), and the 10-item Perceived Stress Scale (PSS-10) (18, 50). Parental satisfaction with pediatric care was assessed using a 10-item scale developed after a review of tools used in various aspects of pediatric care. Only the instruments and variable sets included in the analysis are described below.

#### 2.2.1 European health literacy survey questionnaire (HLS-EU-Q16)

Parental health literacy was assessed using the HLS-EU-Q16, a 16-item short form developed within the European Health Literacy Survey project and derived from the original 47-item HLS-EU questionnaire (46). Items ask respondents to rate how difficult it is to perform common health information tasks across health care, disease prevention, and health promotion. Responses are provided on a four-category scale (“very difficult,” “difficult,” “fairly easy,” “very easy”); “difficult to say/not applicable” responses are treated as missing. Following standard scoring, responses were dichotomized (“very difficult” and “difficult” were coded 0; “fairly easy” and “very easy” were coded 1) and summed to a total score ranging from 0 to 16, calculated only when at least 14 items had valid responses. Total scores were grouped into three levels: inadequate (0–8), problematic (9–12), and sufficient (13–16). Scores were treated as undetermined when more than two items were missing. In the present sample, internal consistency was high (Cronbach's α = 0.88) (51).

#### 2.2.2 e-Health literacy scale (eHEALS)

Self-assessed eHL was measured with the eHEALS, an 8-item instrument that captures respondents' self-reported skills and confidence in using online health information (47, 48). More specifically, the scale addresses perceived ability to identify available online health resources, know where to find them, locate helpful health information on the Internet, evaluate its usefulness and quality, and use such information to answer health questions and support health-related decisions. Thus, the instrument reflects perceived competence in working with electronic health information rather than objective performance in digital information tasks. Items are rated on a 5-point Likert scale from “strongly disagree” to “strongly agree,” with a neutral midpoint. Responses were scored from 1 to 5 and summed to a total score ranging from 8 to 40, with higher scores indicating greater self-assessed ability to use electronic health information for health-related decisions. In the present sample, internal consistency was high (Cronbach's α = 0.90).

#### 2.2.3 Perceived stress scale-10 (PSS-10)

Perceived stress was assessed with the 10-item Perceived Stress Scale (PSS-10), which captures how unpredictable, uncontrollable, and overloaded respondents perceive their lives to have been during the last month (18). The PSS-10 focuses on stress appraisal rather than exposure to specific events, reflecting the extent to which everyday situations are experienced as difficult to manage (52). Items are rated on a 5-point Likert scale (from never to very often). Four positively worded items are reverse-scored, and item scores are summed to a total ranging from 0 to 40, with higher values indicating greater perceived stress. In our sample, the PSS-10 demonstrated a Cronbach's alpha of 0.82, indicating high internal consistency.

#### 2.2.4 Study-specific measure of satisfaction with pediatric care

A study-specific, *ad-hoc* instrument, hereafter referred to as the Parental Cumulative Satisfaction with Pediatric Care Scale (PCS-PCS), was assembled for this study to assess parents' satisfaction with the pediatric care received by their chronically ill child, conceptualized as a cumulative, to-date appraisal across prior contacts, providers, and healthcare settings. An *ad hoc* approach was chosen because, in a pragmatic scoping review of tools available to us in Polish, we did not identify a validated instrument that would both address parents of children with chronic conditions and capture satisfaction as a cumulative, longer-term appraisal of care across multiple contacts, providers, and levels of the health system. In addition, many available questionnaires were relatively lengthy and thus poorly suited to our study design, in which satisfaction with care constituted only one of several constructs assessed in a broader parent survey; therefore, a concise measure was required to minimize respondent burden. In contrast, available standardized measures in Poland have largely focused on satisfaction with inpatient nursing care in pediatric wards or intensive care settings, thus reflecting a single episode of hospital care rather than an integrated, cross-setting trajectory of chronic care (53–55). Likewise, widely used international pediatric experience measures are frequently designed around a defined encounter (e.g., an inpatient stay) and a specific reporting period, which makes them less suitable for evaluating parents' “to date” experiences accumulated over time in chronic disease management (14, 55–57).

The item pool was generated *ad hoc* based on a review of existing questionnaires measuring patient or parent satisfaction and pediatric care experiences, with emphasis on domains repeatedly represented across prior tools. Domain selection was additionally guided by our conceptual interest in examining how parents' satisfaction with care may relate to HL and eHL, particularly through information exchange, shared decision-making, and navigation/coordination of services. We then selected and reformulated content to fit our study aim, i.e., a brief measure reflecting parents' overall appraisal of key aspects of care received so far in relation to their child's chronic condition, rather than satisfaction with a single visit or hospitalization. Drawing on core domains (information and communication, involvement in decision-making, respect and interpersonal aspects, and organization/coordination of care), we formulated 10 items referring to parents' satisfaction with key aspects of pediatric care received to date. Items asked about parents' satisfaction with physicians' information about the child's disease and treatment, physicians' ability to establish rapport with the child, involvement of parents in diagnostic and treatment decisions (including explanations of available options), respect and responsiveness of physicians and other staff, time devoted to the parent and child, coordination/collaboration across different facilities, and the overall quality of care received to date.

Conceptually, in the present study, parental satisfaction was treated as a cumulative evaluative judgment of the pediatric care the child has received so far, whereas patient experience was understood as the set of concrete care processes and interactions on which such judgments are based. For this reason, the content of the PCS-PCS was informed not only by instruments explicitly measuring satisfaction, but also by measures of parent-reported experience, communication, family-centered care, and care coordination. This was a deliberate measurement choice rather than a conceptual conflation of the constructs. Experience-related domains were used to define the main content areas of the scale, while the item wording and response format were designed to capture parents' overall satisfaction with these aspects of care across prior contacts, providers, and settings. Supplementary Table S1 in the Supplementary material documents the source instruments and source items that informed the final wording of the PCS-PCS items.

Each item was rated on a 6-point Likert-type scale, from “strongly disagree” to “strongly agree” with no neutral midpoint; higher scores indicated greater satisfaction. Although the scale was developed pragmatically for this study, its psychometric properties were evaluated in the present sample using exploratory and confirmatory factor analyses and internal consistency indices. The items included in the PCS-PCS, their English wording, and the source instruments that informed their formulation are presented in Supplementary Table S1.

#### 2.2.5 Internet and social media use

Internet use was assessed by asking respondents how much time they typically spend online each day. Categories included in the analysis were: ≤ 30 min, 31–45 min, 46–60 min, 61–90 min, and >90 min. Participants were additionally asked to report their daily duration of social media use, with the following response options included in the analysis: no use, less than 15 min, 15–30 min, 30–60 min, and more than 60 min. These variables were used as broad, frequency-based indicators of general digital engagement. No minimum exposure threshold was imposed because the aim was not to classify respondents according to a predefined criterion for online media use, but to examine whether variation in everyday Internet and social media use was associated with cumulative satisfaction with pediatric care. These indicators were intentionally restricted to daily duration and were not intended to capture other dimensions of online media use, such as device type, communication mode, platform-specific practices, or purpose of use.

#### 2.2.6 Variables characterizing the child and the disease course

Study participants reported the child's age and the duration of their illness, both in years. They were also asked to indicate whether the child had been diagnosed with inflammatory bowel disease (yes/no answers). In addition, participants indicated the number of emergency department visits in the previous year, with three possible answers: 0, 1, or more than 1. Likewise, they reported the number of hospitalizations during the same period, also categorized as 0, 1, or >1.

#### 2.2.7 Sociodemographic variables

Respondents were asked to provide basic demographic data, including age and sex, and to indicate their level of education by selecting one of five categories: below secondary, secondary, post-secondary (non-university), university bachelor's and university master's. Respondents' employment status was assessed using the following categories: employed, self-employed (entrepreneur or farmer), unemployed or part-time employed, and other.

The place of residence was classified into five categories: rural, urban <10,000 inhabitants, urban 10,000–100,000 inhabitants, urban 100,000–500,000 inhabitants, and urban >500,000 inhabitants. Respondents also reported monthly net income per household member by selecting one of the following options: ≤ PLN 1,500, PLN 1,501–2,000, PLN 2,001–3,000, above PLN 3,000, or “refusal to reveal”.

### 2.3 Statistical analysis

All analyses were conducted using IBM SPSS Statistics version 29 (IBM Corp., Armonk, NY, USA) and R version 4.2.0 accessed via the SPSS R integration plug-in. Means and standard deviations were computed for continuous numerical variables, while absolute and relative frequencies were calculated for categorical variables.

Exploratory factor analysis (EFA) was performed in SPSS, and confirmatory factor analyses (CFA) were conducted in R using the lavaan package [version 0.6–17; (58)]. The analytic strategy followed recommended practices for scale development, including separate exploratory and confirmatory phases, appropriate treatment of ordinal indicators, and evaluation of multiple fit indices (59–61).

To support an internal cross-validation of the factor structure and reduce the risk of overfitting, the dataset was randomly split into an exploratory subsample for EFA (≈40%; *n* = 221) and an independent subsample for CFA (≈60%; *n* = 341). The unequal split was chosen to preserve more observations for CFA, where model fit indices and parameter estimates are more sensitive to sample size, while retaining sufficient cases for a stable EFA solution (59). Random allocation was implemented in SPSS using a uniform random variable; cases with missing data on any satisfaction item were excluded listwise from the corresponding analyses.

#### 2.3.1 Exploratory factor analysis

EFA was conducted on the exploratory subsample (*n* = 221) using principal axis factoring (PAF) on the inter-item Pearson correlation matrix. PAF was preferred over principal components analysis because it focuses on common variance and is more appropriate for identifying latent constructs (59). Although the items are ordinal, the use of Pearson correlations in common-factor EFA is considered acceptable when there are several response categories and loadings are strong; this choice is complemented by an ordinal CFA in the validation subsample (62, 63). Sampling adequacy was evaluated using the Kaiser–Meyer–Olkin (KMO) index, and factorability was assessed with Bartlett's test of sphericity. Factor retention was guided by eigenvalues, scree plot, proportion of variance explained, and interpretability. Rotation used direct oblimin (δ = 0), which allows correlated factors and is recommended when factors are expected to correlate (59). For the final one-factor solution, we examined factor loadings and communalities to evaluate item performance and support for a unidimensional total score.

#### 2.3.2 Confirmatory factor analysis

CFA was performed on the independent subsample (*n* = 341) in R using lavaan (58). The primary model specified a single latent factor underlying the 10 items, treated as ordered categorical indicators. The model was estimated with diagonally weighted least squares (DWLS/WLSMV) based on polychoric correlations and robust test statistics, as recommended for Likert-type items (64, 65). One loading (Item1) was fixed to 1.0 to scale the factor, and thresholds for each item were freely estimated. Informed by EFA and item content, we allowed residual covariances between several item pairs to account for local dependencies due to overlapping wording or facets. Model fit was evaluated with χ^2^, Comparative Fit Index (CFI), Tucker–Lewis Index (TLI), Root Mean Square Error of Approximation (RMSEA) with 90% confidence interval and close-fit test, and Standardized Root Mean Square Residual (SRMR), along with their robust counterparts (61, 65, 66). Factor loadings, item-level *R*^2^, and residual covariances were used to characterize item functioning. As a sensitivity analysis, we re-estimated the same one-factor model treating the items as continuous indicators using robust maximum likelihood (MLR), comparing fit indices and standardized parameters across estimators (67). The ordinal DWLS model is reported as primary, and the continuous MLR model is used to demonstrate robustness of the findings.

#### 2.3.3 Hierarchical linear regression

Hierarchical linear regression analysis was conducted to identify predictors of satisfaction with the care. Independent variables were entered into models in four successive blocks to assess their incremental contribution to explained variance. In the first block, sociodemographic variables (age, sex, marital status, place of residence, education, income, and occupational status) were included. The second block introduced variables related to the frequency of physician visits, hospitalizations, child age, illness duration, and IBD diagnosis. In the third block, we added variables characterizing the parents' stress. In the final block, health literacy and e-health literacy scores, and media-use indicators (social media use and Internet use categories) were entered. At each step, changes in R^2^ (ΔR^2^) and corresponding F-statistics were assessed to determine the contribution of the added predictors. Multicollinearity among variables in the models was assessed using variance inflation factors (VIFs) and tolerance values. Unstandardized regression coefficients (B), standard errors (SE), standardized regression coefficients (ß), 95% confidence intervals (95%CI), and *P*-values were reported for independent variables included in the regression models. *P*-values below 0.05 were deemed statistically significant.

## 3 Results

### 3.1 The characteristics of the study sample

Finally, the study was conducted on a sample of 562 parents, of which 82.4% were women (*n* = 463) (Table 1). The mean age of the subjects was 42.0 years (SD = 5.8). A total of 56.9% (*n* = 320) of the participants had a university-level education. Among the respondents, 34.0 % (*n* = 191) lived in rural areas, and 37.9% (*n* = 213) in urban areas with more than 100,000 inhabitants. Parents with sufficient HL levels accounted for 61.7% (*n* = 347) of the sample, while parents with problematic HL levels accounted for 5.5% (*n* = 31). The mean eHL score was 28.5 (SD = 5.0), and the PSS-10 score was 18.7 (SD = 5.8). Satisfaction with care was high, with a mean score of 4.9 (SD = 0,81). Caregivers of children with IBD comprised 66.0% (*n* = 371) of the study sample. In the previous 12 months, 47.2% (*n* = 265) required a hospital stay at least once during this period. The composition of the study sample is detailed in Table 1.

**Table 1:** Characteristics of the study sample.

Variable	Category	%	N
Sex	Male	17.6	99
	Female	82.4	463
Marital status	married or partnership	85.4	480
	Single	4.3	24
	Other	10.3	58
Place of residence	Rural	34.0	191
	urban <10,000	7.5	42
	urban 10,000–100,000	20.6	116
	urban 100,000–500,000	16.5	93
	urban > 500,000	21.4	120
Education	lower than secondary	13.7	77
	secondary	21.2	119
	postsecondary non-university	8.0	45
	university bachelor's	7.1	40
	university master's	49.8	280
Net monthly income per household member^*^	≤ 1,500 PLN	15.3	85
	1,501–2,000 PLN	17.8	101
	2,001–3,000 PLN	19.8	111
	> 3,000 PLN	30.6	172
	refusal to reveal	18.5	87
Vocational status	Employee	51.8	291
	entrepreneur or farmer	17.8	100
	unemployed or part–time job	8.9	50
	Other	21.5	121
Emergency visits in preceding 12 months	no use	62.1	349
	1	18.7	105
	> 1	19.2	108
Hospitalization of the child in preceding 12 months	No	52.8	297
	1	22.6	127
	> 1	24.6	138
Inflammatory bowel disease	No	34.0	191
	Yes	66.0	371
Daily Internet use	≤ 30 min	17.6	99
	31–45 min	14.2	80
	46–60 min	21.5	121
	61–90 min	19.0	107
	>90 min	27.6	155
Daily social media use	no use	13.7	77
	<15 min	21.2	119
	15–30 min	27.9	157
	31–60 min	26.5	149
	> 60 min	10.7	60
Health literacy	Problematic	5.5	31
	Inadequate	17.8	100
	Sufficient	61.7	347
	Undetermined	14.9	84

N, number of respondents; %, percentage of respondents in a given category.

^*^Income categories are reported in PLN, as used in the original questionnaire.

For international orientation, approximate equivalents are as follows: ≤ 1,500 PLN ≈ ≤ EUR 355 / ≤ USD 416; 1,501–2,000 PLN ≈ EUR 355–473 / USD 416–555; 2,001–3,000 PLN ≈ EUR 473–709 / USD 555–832; >3,000 PLN ≈ >EUR 709 / >USD 832, based on Narodowy Bank Polski average exchange rates of March 2, 2026 (Table no. 041/A/NBP/2026; 1 EUR = 4.2304 PLN; 1 USD = 3.6056 PLN). Values are provided for interpretive convenience only.

### 3.2 Exploratory factor analysis (EFA)

EFA was conducted on a random 40% subsample (*n* = 221) to test the dimensionality of the 10-item PCS-PCS. We used principal axis factoring on the Pearson inter-item correlation matrix in IBM SPSS Statistics v.29 with direct oblimin rotation (δ = 0), prioritizing common-factor extraction and empirical retention criteria over principal components (59, 60).

Assumptions were well met (Supplementary Table S2): KMO ≈0.94, Bartlett's test χ^2^ ≈ 2,620 (df = 45), *P* < 0.001, and a small determinant (≈6.5 × 10^−6^), indicating strong but non-singular correlations and supporting a common-factor model (63).

Factor-retention evidence favored a single factor (Supplementary Table S3). The first unrotated factor had an eigenvalue of about 7.99 and explained ~78% of total variance; all remaining eigenvalues were <1, and the scree plot showed a clear elbow after factor 1 (59).

Item statistics aligned with unidimensionality (Supplementary Table S4). Communalities were high (mostly >0.65; several >0.75) and loadings ranged ~0.79–0.99, with Item3 showing the lowest loading and all others >0.80. Rotation did not change interpretation because only one factor was retained. These results support computing a single total score and informed subsequent CFA.

### 3.3 Confirmatory factor analysis (CFA)

CFA was conducted in the remaining 60% subsample (*n* = 341), treating Item1–Item10 as ordered categorical indicators (five response categories). A one-factor model was specified in R (version 4.2.0) using lavaan (version 0.6–17) and DWLS/WLSMV with polychoric correlations and robust (scaled) standard errors and test statistics, as recommended for Likert-type items (64, 65).

Global fit supported the one-factor model (Supplementary Table S5): χ^2^ = 41.82, df = 30, *P* = 0.074; CFI = 1.000; TLI = 1.000; RMSEA = 0.034 (90% CI 0.000–0.057; close-fit test *P*≈0.86); SRMR = 0.02, consistent with common benchmarks (66).

Robust CFI/TLI remained high (0.979/0.968), whereas robust RMSEA was higher (0.102; 90% CI ~0.074–0.130). This pattern can occur when loadings are very strong and residual variances are small, making robust RMSEA numerically sensitive in low–moderate df models (61, 65). Considering the full set of indices and residuals, model fit remained acceptable.

Standardized loadings were high (0.83–0.92; Supplementary Table S6), all *P* < 0.001, with item *R*^2^ values of 0.70–0.85. To address limited, conceptually expected local dependence, a small set of correlated residuals among content-overlapping items was included (Supplementary Table S7). After this adjustment, modification indices did not indicate further meaningful misspecification, and the one-factor structure was retained.

As a secondary check, the same one-factor model was estimated in the CFA subsample (*n* = 341), treating items as continuous, using robust maximum likelihood (MLR) in lavaan (65, 67). Fit was again good (Supplementary Table S5): χ^2^ = 75.94, df = 30, *P* < 0.001; CFI = 0.987; TLI = 0.980; RMSEA = 0.067 (90% CI 0.048–0.086); SRMR = 0.020. Robust indices were also strong (robust CFI = 0.993; robust TLI = 0.989; robust RMSEA = 0.049, 90% CI 0.008–0.078; close-fit test *P*≈0.49). Loadings and explained variance were consistent with the ordinal model (Supplementary Table S6), and the same residual correlations were modest in size (Supplementary Table S7).

Together, the ordinal and continuous CFAs converge on the same conclusion: PCS-PCS is essentially unidimensional, with high loadings and substantial explained item variance. The ordinal specification is retained as the primary model, while the continuous model indicates that conclusions are not estimator-dependent.

### 3.4 Determinants of satisfaction with pediatric care

In step 1, sociodemographic and socioeconomic variables produced a statistically significant model, F (19, 413) = 2.24, *P* = 0.002, explaining 9.4% of variance (*R*^2^ = 0.094; adjusted *R*^2^ = 0.052). Overall, the effects in this block were modest, with selected differences by marital status, residence, education, and income. In step 2, adding clinical variables (physician visits, hospitalizations, child age, illness duration, IBD) did not meaningfully improve explained variance (*R*^2^ = 0.106; ΔR^2^ = 0.012; F-change (7, 406) = 0.79, *P* = 0.598), and none of these clinical indicators showed an independent association at the conventional alpha level. In step 3, adding perceived stress significantly improved model performance (*R*^2^ = 0.154; adjusted *R*^2^ = 0.097; ΔR^2^ = 0.048; F-change (1, 405) = 22.91, *P* < 0.001). Higher stress was associated with lower satisfaction (B = −0.03; 95%CI:−0.05 to -0.02).

In step 4, HL, eHL, and media-use indicators were added, yielding a further incremental gain (ΔR^2^ = 0.049; F-change (12, 393) = 2.00, *P* = 0.023) and a final model explaining 20.2% of variance (F (39, 393) = 2.55, *P* < 0.001; *R*^2^ = 0.202; adjusted *R*^2^ = 0.123). In this final model, stress remained a robust negative predictor (B = −0.03; 95%CI:−0.04 to−0.02). Inadequate HL (vs sufficient HL) was associated with lower satisfaction (B = −0.64; 95%CI:−0.98 to−0.31). Several covariates retained independent associations (married/partnered: B = −0.34; 95%CI:−0.59 to−0.10; city >500,000 residents: B = −0.25; 95%CI:−0.47 to−0.04; university bachelor's education: B = 0.40; 95%CI: 0.09 to 0.71; income ≤ 1,500 PLN: B = −0.37; 95%CI:−0.61 to−0.12). Other HL categories, eHL, and media-use indicators were not statistically significant in the adjusted model. A summary of the model is provided in Table 2, with detailed coefficients in Supplementary Table S8.

**Table 2:** Results of hierarchical linear regression predicting parental satisfaction with pediatric care.

Variable	Categories of variables	Model 1 β	Model 2 β	Model 3 β	Model 4 β
Age		0.04	0.02	0.01	−0.01
Sex	male#				
	Female	−0.01	−0.01	0.03	0.02
Marital status	other#				
	married or in partnership	0.12^*^	−0.10	0.13^*^	0.15^**^
	Single	−0.09	−0.07	−0.07	−0.08
Place of residence	rural#				
	urban <10,000	0.02	0.02	0.02	0.02
	urban 10,000–100,000	−0.07	−0.06	−0.05	−0.05
	urban 100,000–500,000	−0.07	−0.04	−0.04	−0.05
	urban >500,000	0.11^**^	0.10^**^	0.12^*^	−0.12
Education	university Master's#				
	lower than secondary	0.15	0.16	0.13	0.11
	secondary	0.09	0.08	0.07	0.05
	post-secondary non-university	0.08	0.07	0.05	0.04
	university Bachelor's	0.15^**^	0.14^**^	0.13^*^	0.12^*^
Net monthly income per household member	2,001–3,000 PLN#				
	≤ 1,500 PLN	0.20^**^	0.19^**^	0.16^**^	0.18^**^
	1,501–2,000 PLN	−0.08	−0.07	−0.07	−0.08
	>3,000 PLN	0.02	0.02	0.01	0.001
	refusal to reveal	−0.04	−0.03	−0.03	−0.04
Vocational status	employee #				
	entrepreneur or farmer	0.07	0.07	0.05	0.06
	unemployed or part-time job	0.03	0.04	0.06	0.06
	Other	−0.04	−0.03	−0.04	−0.05
Age of a child			0.04	0.02	0.02
Duration of disease			−0.05	−0.05	−0.04
Emergency services in the preceding 12 months	no use#				
	1		−0.04	−0.03	−0.02
	>1		−0.02	−0.02	−0.004
Hospitalization of the child in the preceding 12 months	no#				
	1		−0.01	0.01	0.02
	>1		−0.07	−0.03	−0.02
Inflammatory bowel disease	no#				
	yes		0.06	0.04	0.03
PSS-10 score				0.24^***^	0.22^***^
Daily Internet use	>90 min#				
	≤ 30 min				0.06
	31–45 min				0.01
	46–60 min				−0.09
	61–90 min				0.01
Daily social media use	15–30 min #				
	<15 min				0.03
	31–60 min				0.04
	>60 min				0.07
eHL					−0.06
HL	sufficient #				
	Inadequate				0.18^***^
	Problematic				−0.04
	Undetermined				−0.06
R^2^		0.094	0.106	0.154	0.202
DR^2^		0.094^**^	0.012	0.048^***^	0.049^*^
F (df1, df2)		2.24 (19, 413)	0.79 (7, 406)	22.91 (1, 405)	2.00 (12, 393)

PSS, perceived stress scale; eHL, e-health literacy; HL, health literacy; min, minutes; β – standardized regression coefficient; R^2^ coefficient of determination; ΔR, change of R^2^; F, test for the change in R^2^; #, reference category; ^*^ – *P* < 0.05, ^**^ – *P* < 0.01, ^***^ – *P* < 0.001.

## 4 Discussion

### 4.1 Summary of findings

In this study, we examined how parents' cumulative satisfaction with pediatric care for a child with a chronic condition relates to parental resources, psychosocial context, and indicators of illness course and service use. We define satisfaction as a global appraisal formed across repeated health system contacts, reflecting communication, continuity, coordination, and responsiveness over time (7–9). Unlike patient experience measures that document what occurred, satisfaction is evaluative and shaped by expectations and personal context, so it is not a direct measure of specific processes.

To capture this construct, we used a concise, study-specific Parental Cumulative Satisfaction with Pediatric Care scale. In this sample, EFA and CFA supported an essentially unidimensional structure with strong internal consistency and good factorial validity, consistent with a single latent factor. Because results come from one dataset, they provide internal support rather than full validation; external validity should be tested against independent measures of care experience, communication quality, and care coordination. A small set of conceptually plausible correlated residuals improved local fit without changing interpretation and likely reflects minor item overlap.

Hierarchical regression showed that sociodemographic background accounted for a modest share of variance, and illness/service-use indicators added little. The main gains came from perceived stress and HL; overall, the final model explained ~20% of variance in the present sample. This mirrors appraisal-based stress work, where perceived overload and low control can shape global evaluations of support (18, 19). This level of explained variance also indicates that substantial heterogeneity in satisfaction remains unaccounted for, pointing to the likely importance of unmeasured determinants such as specific experiences with access, communication, and coordination, as well as family preferences and expectations.

In the fully adjusted model, higher perceived stress predicted lower satisfaction. Similar associations between caregiver strain or distress and poorer care appraisals have been reported in pediatric chronic illness samples (4, 68). Given the cross-sectional, self-report design, directionality is uncertain. Inadequate HL also predicted lower satisfaction, in line with evidence that limited HL affects navigation and communication and increases management burden (24, 32). By contrast, eHL and general digital media use were not independently associated, consistent with research that broad online activity is a weak proxy for mechanisms through which digital resources may shape care evaluations (34, 35). The null findings for digital variables are therefore meaningful and indicate that broad markers of online engagement do not translate straightforwardly into more positive or more negative global evaluations of pediatric care when key psychosocial and literacy factors are taken into account.

Sociodemographic differences persisted, indicating that satisfaction is socially patterned. Lower satisfaction among lower-income parents is consistent with evidence that disadvantage can constrain access and increase practical costs, while differences by urban residence and education may reflect variation in service organization and expectations (32). Because access barriers, waiting times, and facility-level characteristics were not measured, these interpretations remain contextual explanations rather than demonstrated mechanisms. The limited contribution of illness and utilization indicators suggests that between-family differences in cumulative satisfaction may relate more to parental resources and appraisal context than to the clinical markers available here, and that continuity and coordination may matter more than contact volume *per se* (6, 15). This inference should be cautious because continuity or coordination were not measured directly and illness and utilization indicators may be too coarse (e.g., disease activity, severity, complications) or operate non-linearly or in subgroups.

### 4.2 Health literacy

In the fully adjusted model, lower HL remained associated with lower cumulative satisfaction with pediatric care. This is consistent with the view that satisfaction reflects the fit between the informational and organizational demands of care and families' capacity to understand, appraise, and use health information in everyday management (21, 69). In pediatric chronic care, parents coordinate treatment and monitoring. Parental HL can support communication, understanding recommendations, and navigation across services, including interpreting written materials and completing administrative tasks. Systematic reviews link limited HL to poorer outcomes and difficulties engaging with care (24, 69). Parent-focused studies also associate lower HL with perceived barriers and less participation in shared decision-making, which may reduce the sense that care is responsive and collaborative (25). Limited HL has also been linked to medication administration errors, which can add frustration and uncertainty in long-term regimens (28).

The association persisted after adjustment for sociodemographic characteristics and perceived stress, indicating explanatory value beyond education or psychosocial strain. This does not imply a direct causal pathway from HL to satisfaction. HL may also mark broader resources (e.g., self-efficacy, communication confidence, prior experience navigating the health system) and may partly mediate socioeconomic gradients in satisfaction. Clinically, the findings suggest that cumulative satisfaction depends in part on whether care is understandable, navigable, and usable for parents with different literacy profiles. A conservative implication is to reduce informational burden and improve clarity and navigability, rather than assuming that raising HL alone would increase satisfaction. Clear communication, confirmation of understanding, and structured coordination support may be particularly important in long-term pediatric care pathways.

### 4.3 E-health literacy

In contrast to HL, eHL showed no independent association with cumulative satisfaction in the adjusted model. This null finding is informative: after accounting for HL and stress, perceived ability to use online health resources does not appear to shape how parents evaluate pediatric care. Conceptually, eHL reflects confidence in finding and using web-based health information (36). Cumulative satisfaction, however, is mainly an appraisal of care delivery and system responsiveness. Online information skills may influence satisfaction only when they change clinical interactions, for example, by improving question formulation, shared understanding, or care planning. Our results are consistent with substantial overlap between eHL, broader HL, and sociodemographic resources, leaving little unique variance for eHL once these factors are modeled together. Measurement issues may also contribute. The eHEALS captures perceived skills for using online health information, but it may not reflect current information ecologies, including social media consumption, algorithmically curated content, and the need to appraise misinformation, which could weaken associations with global satisfaction outcomes. Prior work suggests that eHL may relate more strongly to proximal outcomes (e.g., shared decision-making, wellbeing) than to global satisfaction, and that effects can be context- and outcome-specific (37). In chronic pediatric care, parents may use online information to complement or verify professional advice, yet this may not systematically shift overall care appraisals when access, continuity, and communication within the health system are the dominant drivers of satisfaction.

### 4.4 Internet and social media use

Measures of Internet use and social media use were not independently associated with cumulative satisfaction in the full model. This suggests that frequency of digital engagement is a poor proxy for how parents evaluate care. Digital engagement varies in content and purpose: parents may seek practical information, peer support, navigation, or emotional coping, and benefits can coexist with burdens, producing a net association close to zero. Scoping reviews show that social media is commonly used for child health information and support, yet consequences include both empowerment and greater exposure to conflicting information or distressing narratives (35). Reviews of digital tools for shared self-management in childhood long-term conditions likewise emphasize heterogeneity in purpose, quality, and integration with clinical care, which can weaken any simple link between use frequency and satisfaction (34).

From a health-services perspective, the null finding reinforces that satisfaction is not determined by information availability alone. When service organization, access, or communication are suboptimal, more online searching may not improve experience; when pathways function well, online engagement may be common but not decisive for global satisfaction. Because our indicators captured frequency rather than purpose, content, or quality, opposing mechanisms may cancel out at the aggregate level. More broadly, review literature indicates that parental online health information seeking is heterogeneous with respect to motivations, trust judgments, and the ways retrieved information is used in decision-making for the child, while digital tools in childhood chronic care also vary widely in purpose, content, and integration with clinical care (35, 70). Against this background, measures based only on time spent online provide only a partial picture of digital engagement. Future work should use more specific measures of online behaviors and assess whether online resources are aligned with clinical communication and care planning, rather than relying on indicators based on time alone.

### 4.5 Perceived stress

Perceived stress was a robust correlate of lower cumulative satisfaction in the fully adjusted model. This pattern aligns with appraisal-based stress models in which perceived overload and limited controllability shape evaluations of demanding situations (18, 19). Parenting a child with a chronic condition involves sustained practical and emotional demands, and meta-analyses show elevated parenting stress across pediatric chronic illnesses (4, 5). Stress is also linked to mental health and wellbeing, which can influence how healthcare encounters are perceived and remembered over time (3). Stress may lower satisfaction through several pathways: increasing sensitivity to barriers, reducing tolerance for coordination failures or unclear communication, and amplifying the perceived impact of delays or inconsistencies. It may also indicate unmet needs for support and reassurance that parents attribute to the health system when care feels fragmented or insufficiently family-centered.

The persistence of this association after adjustment suggests that cumulative satisfaction reflects not only technical care delivery but also the psychosocial context in which care is received. Because perceived stress here captures general recent stress rather than condition-specific caregiving strain, it may reflect broader life demands that co-occur with pediatric chronic illness, and the association should not be attributed to caregiving burden alone. The findings support integrating psychosocial screening and support in chronic care pathways and using communication strategies that acknowledge uncertainty, provide anticipatory guidance, and reduce avoidable informational burden, which may be especially important for highly stressed parents.

### 4.6 Disease course indicators and child age

Indicators describing the child's illness course, including child age and selected utilization markers, did not contribute meaningfully to cumulative satisfaction in the full model. This null pattern suggests that parents' global appraisal of pediatric care is not driven primarily by disease descriptors or contact frequency, at least as captured by these measures. Satisfaction and patient experience are commonly understood as reflections of care processes and interactions, such as communication quality, continuity, and responsiveness, rather than direct proxies for disease severity (7–9). In pediatric chronic care, families may adapt to stable patterns of follow-up, and differences in utilization volume may not map onto perceived quality if coordination and communication remain similar across groups. The lack of association is also consistent with evidence that parents' experience of care for complex conditions depends strongly on continuity and coordination across services, dimensions that are not reducible to the number of visits or hospitalizations (6, 15, 40).

At the same time, the finding does not imply that the clinical course is irrelevant to families' lives; rather, it indicates that its relationship with satisfaction is likely indirect and mediated by how services respond to illness-related needs. Condition-specific barriers to care in pediatric inflammatory bowel disease have been documented from patient, parent, and provider perspectives, pointing to practical constraints and system-level bottlenecks that may shape experience beyond the illness course itself (39). The present results suggest that such experiential factors may not be captured by broad clinical descriptors and that qualitative aspects of service delivery remain the more proximal determinants of cumulative satisfaction.

Alternative explanations should also be considered: the clinical and utilization variables may not adequately represent disease severity or activity, and relationships may be non-linear or differ across subgroups. For example, effects of child age could be non-linear across developmental stages, and a linear specification may therefore underestimate age-related differences in satisfaction.

### 4.7 Sociodemographic factors

Several sociodemographic and socioeconomic variables remained associated with satisfaction in the full model, indicating that cumulative satisfaction is socially patterned. Lower satisfaction among parents with fewer economic resources is consistent with evidence that disadvantage can increase the practical costs of care, limit access to supportive services, and amplify the consequences of system fragmentation (6, 25). Differences by place of residence may reflect variation in service organization and continuity, as well as in expectations and perceived responsiveness, in high-volume urban settings, although cross-sectional data cannot clarify mechanisms.

With respect to digital access, the meaning of place of residence is likely to be context-dependent. In settings with weaker digital infrastructure, residence may more directly reflect barriers to Internet access and use, including broadband availability and digital inclusion (71, 72). In contemporary Poland, however, household Internet access is high overall, and recent official statistics suggest that urban-rural differences in basic access are relatively small, although some differences in broadband access and related aspects of digital connectivity may still persist (73). Therefore, the association observed here for place of residence should not be interpreted narrowly as a proxy for basic Internet availability alone, but may also reflect differences in service organization, expectations, travel burden, or other contextual factors.

Educational differences may similarly reflect both resources for navigating care and differences in expectations about communication, partnership, and coordination. These gradients matter because patient experience has been linked to system-level effectiveness and safety (12). The persistence of socioeconomic differences after adjustment suggests that reducing inequities in cumulative satisfaction may require service-level strategies that lower informational and organizational burden, strengthen care coordination, and support parents with fewer resources. Such approaches align with continuity-oriented chronic care and family-centered coordination frameworks addressing navigation across settings and providers (6, 15, 40). Nonetheless, because we did not collect facility-level or system-level indicators, the present results should be interpreted as evidence of social patterning rather than as evidence identifying where, within the system, these differences arise.

### 4.8 Practical implications

The findings suggest that, in chronic pediatric care, parents' cumulative satisfaction is shaped more by parental resources and psychosocial context than by the illness-course and contact-volume indicators included here. Practically, this supports prioritizing interventions that reduce informational and navigational burden, improve communication clarity, and strengthen coordination across settings, consistent with satisfaction as a global evaluation of care over time (7, 9, 15).

The independent association between limited HL and lower satisfaction implies that services should be usable across the full HL spectrum, rather than expecting parents to compensate for complex information or fragmented pathways. Actionable steps include plain-language communication, structured confirmation of understanding (e.g., teach-back), and clear written materials for long-term monitoring and treatment plans (21, 32, 69). Because families often coordinate across clinics, hospitals, diagnostics, and ancillary services, predictable pathways and explicit navigation support may benefit all families and may especially help those with fewer resources.

Perceived stress was negatively associated with satisfaction, indicating that psychosocial strain is relevant for how care is appraised over time. Without assuming causality, chronic care pathways should include routine identification of high-stress families and clear routes to support. Brief, structured conversations that validate uncertainty, provide anticipatory guidance, and clarify who to contact may reduce the perceived “coordination load,” consistent with appraisal-focused stress models (18, 19, 52).

Null findings for eHL and for general Internet/social media use suggest that higher online activity alone is unlikely to improve satisfaction. A practical approach is to recommend trustworthy, diagnosis-relevant resources and explicitly invite discussion of online information during consultations to connect information seeking with shared understanding and care planning (35, 36).

Socioeconomic gradients also support an equity focus: simplifying administrative steps, improving appointment logistics, and standardizing coordination functions may yield the greatest benefit for lower-income families facing higher opportunity costs (6, 32).

### 4.9 Strengths of the study

An important strength of this study is that it examines parental satisfaction with pediatric care from a cumulative chronic-care perspective, rather than restricting the assessment to a single hospitalization or outpatient visit. This approach is particularly relevant for parents of children with chronic gastrointestinal diseases, whose evaluations of care are shaped by repeated contacts with the healthcare system, the need for coordination across settings, and the long-term practical implementation of medical recommendations in everyday family life. Another strength is the multicenter design, with data collected in five university gastroenterology centers, which increased the diversity of care contexts represented in the sample and strengthened the clinical relevance of the findings within specialist pediatric care.

A further strength is the combination of instrument development with substantive analysis. Although the PCS-PCS was developed specifically for this study, its internal psychometric performance was examined in separate exploratory and confirmatory stages, including CFA for ordinal items. This provides a stronger basis for using the total score than relying solely on face validity. In addition, the hierarchical regression approach enabled assessment of the incremental contribution of successive blocks of predictors and examination of HL, eHL, perceived stress, and digital engagement, while accounting for sociodemographic and illness-related variables. This analytic strategy allowed us to identify which factors retained independent explanatory value and to distinguish more proximal psychosocial and literacy-related correlates from broader background and clinical characteristics.

### 4.10 Limitations

The cross-sectional design precludes causal inference and limits conclusions about directionality. Associations between perceived stress and satisfaction, and between health literacy and satisfaction, should therefore be interpreted as correlational, with plausible bidirectionality.

Most variables, including satisfaction, stress, HL, eHL, and digital media use, were self-reported at a single time point. This may introduce common method variance and shared response tendencies, and some illness-course and utilization indicators may be affected by recall error when parents summarize longer periods.

The study-specific satisfaction scale demonstrated strong internal psychometric performance in this sample, supporting an essentially unidimensional score. However, evidence for external validity remains limited: the instrument was not benchmarked against independent measures of care experience domains (e.g., communication quality, care coordination, access), test–retest reliability was not assessed, and measurement invariance across key subgroups was not evaluated. The use of a small number of correlated residuals improved local fit but may reflect item content overlap; replication in an independent sample would strengthen confidence in the model.

Digital determinants were operationalized broadly. eHEALS captures self-perceived competence in using online health information but may not fully reflect contemporary digital information environments, and frequency-based indicators of Internet and social media use do not capture purpose, content, or source credibility. They also do not reflect other dimensions of online media use, such as device type, communication mode, or platform-specific practices. They were also not designed to classify respondents according to a minimum threshold of online media use. These measurement choices may partly contribute to the observed null associations for digital variables and may also limit comparability with studies using more restrictive or behavior-specific definitions of online media exposure. This limitation is consistent with review literature showing that both parental online health information seeking and eHL are heterogeneous constructs that are defined and measured differently across studies (70, 74).

Clinical and utilization variables may also have been too coarse to capture meaningful variation in disease burden. Without direct indicators of disease activity or severity, null findings for illness-course markers should be interpreted cautiously. Moreover, utilization indicators such as visit frequency are ambiguous and do not reflect qualitative care processes that are central to satisfaction (7, 9).

The final model explained a modest share of variance in satisfaction, suggesting that additional determinants were not measured, such as access barriers, waiting times, perceived continuity and coordination, and family-centered communication (6, 15). Finally, generalizability depends on the study context; replication in other settings and with more detailed care experience and clinical measures is needed to assess the stability and transportability of the observed associations.

## 5 Conclusions

Cumulative parental satisfaction with pediatric care in a chronic illness context appears to be shaped primarily by parents' psychosocial and informational resources rather than by the included markers of disease course and service intensity. The pattern of associations supports a view of satisfaction as an evaluative outcome that is sensitive to how manageable care feels to families, including whether information is understandable and actionable, and whether the broader situation is experienced as overloaded or difficult to control.

These findings underscore that improving families' experience of long-term pediatric care likely requires attention to the usability of communication and care pathways across the full spectrum of HL, alongside routine recognition of psychosocial strain. At the same time, the absence of independent associations for eHL and general digital media use suggests that digital engagement *per se* is not a reliable lever for satisfaction unless connected to specific, high-quality resources and integrated into clinical communication and care coordination.

## Data Availability

The datasets presented in this study can be found in online repositories. The names of the repository/repositories and accession number(s) can be found below: Zenodo doi: 10.5281/zenodo.18938607; https://zenodo.org/uploads/18938607.
