# Supplementary material for: The relationship between health literacy and satisfaction with medical care in parents of children with chronic gastrointestinal diseases
Source: Front Public Health. 2026 Apr 29;14:1828560. doi: 10.3389/fpubh.2026.1828560 (PMC13168047; doi:10.3389/fpubh.2026.1828560)
Supplement: Supplementary file 1 [file Data_Sheet_1.pdf]

Supplementary Material

Tabela S1. Origin of items used in the Parental Cumulative Satisfaction Pediatric Care Scale (PCS-PC)

Item (Polish)	Item (English)	Source instrument	Source item	Reference
1.Z zakresu i jasności przekazywanych przez lekarzy informacji dotyczących choroby Pana/Pani dziecka	1. Regarding the scope and clarity of information provided by doctors about your child's illness.	Child Zufriedenheit in der Arztpraxis (The Child ZAP)	Information received about your child's illness?<br>Comprehensibility / clarity of information?<br>(Parents information)	Bitzer et al., 2012
		EMPATHIC	We were given clear information about our child's disease<br>(Information)	Latour et al., 2011
		Child Hospital Consumer Assessment of Healthcare Providers and Systems (Child HCAHPS®)	During this hospital stay, how often did your child's doctors explain things to you in a way that was easy to understand?<br>(Communication with parent)	Toomey et al., 2015
		Family-Centered Care Assessment tool (FCCA)	My child's health care provider talks with me using words I understand.	Wells et. al., 2015
		Pediatric Provider Communication Skills Assessment Scale	I try to talk to the child in a simple and understandable language.	Boztepe et al., 2023
		Kalamazoo Essential Elements Communication Checklist (KEECC)	Explains using words that are easy for patient to understand<br>(Shares information)	Makoul, 2001
2. Z zakresu i jasności przekazywanych przez lekarzy informacji dotyczących planowanego leczenia i działania przepisanych Pana/Pani dziecku leków	2. Regarding the scope and clarity of information provided by doctors about the planned treatment and the effects of medications prescribed for your child.	The Child Zufriedenheit in der Arztpraxis (The Child ZAP)	Information about planned treatments for your child?<br>Information on the effects of prescribed medications?<br>Comprehensibility / clarity of information?<br>(Parents information)	Bitzer et al.,2012
		EMPATHIC	The doctor clearly informed us about the consequences of our child's treatment<br>We received understandable information about the effects of the drugs<br>(Information)	Latour et al., 2011

		Child Hospital Consumer Assessment of Healthcare Providers and Systems (Child HCAHPS®)	During this hospital stay, how often did your child's doctors explain things to you in a way that was easy to understand?<br><br>During this hospital stay, how often did providers keep you informed about what was being done for your child? (Communication with parent)	Toomey et al., 2015
		Family-centered care assessment tool (FCCA)	My child's health care provider has a way to help my child understand medical tests and treatments before he or she does them	Wells et al., 2015
		Patient Satisfaction Questionnaire (PSQ-18)	Doctors are good about explaining the reason for medical tests<br>When I go for medical care, they are careful to check everything	Marshall & Hays, 1994
		Pediatric Provider Communication Skills Assessment Scale	I inform the child/ family about the treatment process.	Boztepe et al., 2023
3. Z umiejętności nawiązywania kontaktu przez lekarzy z Pana/Pani dzieckiem	3. Regarding doctors' ability to establish rapport with your child.	Child Hospital Consumer Assessment of Healthcare Providers and Systems (Child HCAHPS®)	During this hospital stay, how often did your child's doctors listen carefully to you? (Communication with parent)<br>During this hospital stay, how often did your child's doctors listen carefully to your child? (Communication with child)	Toomey et al., 2015
		Family-Centered Care Scale (FCCS)	Help me feel welcomed	Curley et al., 2013
		The CARE Measure	Really listening<br>(paying close attention to what you were sayings; not<br>looking at the notes or computer as you were talking)	Merce et al., 2004
		Pediatric Provider Communication Skills Assessment Scale	I make eye contact while talking to the child.<br>No matter what the subject is, I listen to the child patiently.	Boztepe et al., 2023
		Kalamazoo Essential Elements Communication Checklist (KEECC)	Uses tone, pace, eye contact, and posture that show care and concern (Builds a Relationship)	Makoul, 2001
4. Ze stopnia i sposobu angażowania Pana/Panią w decyzje dotyczące badań i	4. Regarding the degree and manner of involving you in decisions concerning your	The Child Zufriedenheit in der Arztpraxis (The Child ZAP)	He/She asks me which option I prefer for my child.<br>I am satisfied with the extent to which I am involved in decision making.<br>(Parents decision- making)	Bitzer et al.,2012

leczenia Pana/Pani dziecka	child’s tests and treatment.	EMPATHIC	We were actively involved in decision-making on care and treatment of our child (Parental participation)	Latour et al., 2011
		Family-Centered Care Scale (FCCS)	Ask me how I want to participate in my child’s care Treat me as a valued team member when planning my child’s nursing care	Curley et al., 2013
		Parent-physician communication and decision-making studies	"Was your perspective about your child's treatment and care considered?" (Shared decision-making assessment).	Aarthun et al., 2018
		Family-centered care assessment tool (FCCA)	My child’s health care provider supports me in the role that I want to take in making decisions about my child’s health care.	Wells et al., 2015
		The Care Measure	Making a plan of action with you (discussing the options, involving you in decisions as much as you want to be involved; not ignoring your views)	Merce et al., 2004
5. Z wyjaśnień dotyczących możliwości i sposobów badań, leczenia i zapobiegania chorobie Pana/Pani dziecka	5. Regarding explanations about the options and methods of examination, treatment, and prevention of your child’s illness.	The Child Zufriedenheit in der Arztpraxis (The Child ZAP)	Information on what you can do to promote your child's recovery? (Parents information)<br>He/She informs me of different options (e.g., for examination or treatment of my child).<br>He/She asks me which option I prefer for my child.<br>(Parents decision- making)	Bitzer et al.,2012
		Family-centered care assessment tool (FCCA)	My child’s health care provider talks with me about promoting my child’s overall health and well-being.	Wells et al., 2015
		The Parent Health Literacy Questionnaire (HLQ-Parent)	"The doctor explained the different options for treatment"	Walh et al., 2024
		The Care Measure	Helping you to take control (exploring with you what you can do to improve your health yourself; encouraging rather than “lecturing” you)	Merce et al., 2004
6.Z szacunku okazywanego Panu/Pani i Pana/Pani dziecku przez lekarzy i inny	6. Regarding the respect shown to you and your child by doctors and other medical staff.	The Child Zufriedenheit in der Arztpraxis (The Child ZAP)	Understanding of your child?<br>Treating your child as an individual?<br>Patience with your child?<br>(Child- interaction)	Bitzer et al.,2012
		EMPATHIC	The team showed respect for our child and for us	Latour et al., 2011

personel medyczny			(Professional attitude)	
		Child Hospital Consumer Assessment of Healthcare Providers and Systems (Child HCAHPS®)	During this hospital stay, how often did your child's doctors treat you with courtesy and respect?<br>(Communication with parents)	Toomey et al., 2015
		The Care Measure	Showing care and compassion (seeming genuinely concerned, connecting with you on a human level; not being indifferent or "detached")	Merce et al., 2004
		Pediatric Provider Communication Skills Assessment Scale	I always communicate with the child in an affectionate manner and language	Boztepe et. al., 2023
7. Z reakcji personelu medycznego na wyrażane przez Pana/Panią i Pana/Pani dziecko potrzeby i prośby	7. Regarding the medical staff's response to the needs and requests expressed by you and your child.	The Child Zufriedenheit in der Arztpraxis (The Child ZAP)	Empathy for your child?<br>Taking your child seriously?<br>(Child- interaction)	Bitzer et al.,2012
		Family-Centered Care Scale (FCCS)	Help me to feel well-cared-for<br>Help my child to feel well-cared-for	Curley et al., 2013
		EMPATHIC	Our own needs were well responded to by the doctors<br>Our child's needs were well responded to by the doctors<br>(Care and cure)	Latour et al., 2011
		Family-centered care assessment tool (FCCA)	I feel comfortable letting my child's health care provider know when I disagree with recommendations for my child's health care.	Wells et al., 2015
		Patient Satisfaction Questionnaire (PSQ-18)	My doctors treat me in a very friendly and courteous manner	Marshall & Hays, 1994
8. Z czasu poświęcanego przez personel medyczny Panu/Pani i Pana/Pani dziecku	8. Regarding the time devoted by medical staff to you and your child.	Patient Satisfaction Questionnaire (PSQ-18)	Doctors usually spend plenty of time with me	Marshall & Hays, 1994
		Kalamazoo Essential Elements Communication Checklist (KEECC)	Allows patient to complete opening statement without interruption<br>(Open Discussion)	Makoul, 2001
9. Ze współpracy pomiędzy lekarzami i pracownikami ochrony zdrowia z różnych placówek	9. Regarding cooperation between doctors and healthcare professionals from different facilities.	The Child Zufriedenheit in der Arztpraxis (The Child ZAP)	Collaboration with other medical facilities?"<br>(Professional competence)	Bitzer et al., 2012
		EMPATHIC	The doctors and nurses worked closely together<br>(Care and cure)	Latour et al., 2011
		SEIPS 2.0 framework (Systems Engineering Initiative for Patient Safety); Inter-organizational communication studies	"Effective communication between different care providers regarding your child's treatment" (SEIPS framework).	Holden et al., 2021; Carayon et al., 2020

10. Z jakości opieki medycznej, którą do tej pory uzyskało Pana/Pani dziecko	10. Regarding the quality of medical care your child has received so far.	The Child Zufriedenheit in der Arztpraxis (The Child ZAP)	How satisfied are you with the quality of care by this pediatrician?<br>(Global items)	Bitzer et al., 2012
		EMPATHIC	The team worked efficiently (Organization)	Latour et al., 2011
		Patient Satisfaction Questionnaire (PSQ-18)	*The medical care I have been receiving is just about perfect*	Marshall & Hays, 1994

The list of full references referred to in Table S1.

1. Bitzer, E. M., Volkmer, S., Petrucci, M., Weissenrieder, N., & Dierks, M. L. (2012). Patient satisfaction in pediatric outpatient settings from the parents' perspective: The Child ZAP: A psychometrically validated standardized questionnaire. BMC Health Services Research, 12(1), 347. doi.org/10.1186/1472-6963-12-347
2. Latour, J. M., Van Goudoever, J. B., Duivenvoorden, H. J., Albers, M. J. I. J., Van Dam, N. A. M., Dullaart, E., Van Heerde, M., De Neef, M., Verlaat, C. W. M., Van Vught, E. M., & Hazelzet, J. A. (2011). Construction and psychometric testing of the EMPATHIC questionnaire measuring parent satisfaction in the pediatric intensive care unit. Intensive Care Medicine, 37(2), 310–318. doi.org/10.1007/s00134-010-2042-y
3. Toomey, S. L., Zaslavsky, A. M., Elliott, M. N., Gallagher, P. M., Fowler, F. J., Klein, D. J., Shulman, S., Ratner, J., McGovern, C., LeBlanc, J. L., & Schuster, M. A. (2015). The development of a pediatric inpatient experience of care measure: Child HCAHPS. *Pediatrics, 136*(2), 360–369. doi.org/10.1542/peds.2015-0966
4. Curley, M. A. Q., Hunsberger, M., & Harris, S. K. (2013). Psychometric evaluation of the Family-Centered Care Scale for pediatric acute care nursing. *Nursing Research, 62*(3), 160–168. doi:10.1097/NNR.0b013e318286d64b
5. Wells, N., Bronheim, S., Zyzanski, S., & Hoover, C. (2015). Psychometric evaluation of a consumer-developed family-centered care assessment tool. *Maternal and Child Health Journal, 19*(9), 1899–1909. doi.org/10.1007/s10995-015-1709-y
6. Wahl, A. K., Hermansen, Å., Tschamper, M. B., Osborne, R. H., Helseth, S., Jacobsen, R., & Larsen, M. H. (2024). The Parent Health Literacy Questionnaire (HLQ-Parent): Adaptation and validity testing with parents of children with epilepsy. Scandinavian Journal of Public Health, 52(1), 39–47. doi.org/10.1177/14034948221123436
7. Aarthun, A., Øymar, K., & Aagaard, H. (2018). Parental involvement in decision-making about their child's health and care. *Journal of Child Health Care, 22*(3), 388–397. doi:10.1002/nop2.18
8. Marshall, G. N., & Hays, R. D. (1994). *The Patient Satisfaction Questionnaire Short-Form (PSQ-18)* (Vol. 7865). Santa Monica, CA: RAND Corporation.
9. Mercer, S. W., Maxwell, M., Heaney, D., & Watt, G. C. (2004). The Consultation and Relational Empathy (CARE) measure: Development and preliminary validation and reliability of an empathy-based consultation process measure. *Family Practice, 21*(6), 699–705. doi.org/10.1093/fampra/cmh621
10. Holden, R. J., & Carayon, P. (2021). SEIPS 101 and seven simple SEIPS tools. *BMJ Quality & Safety, 30*(11), 901–910. doi.org/10.1136/bmjqs-2020-012538
11. Carayon, P., Wooldridge, A., Hoonakker, P., Hundt, A. S., & Kelly, M. M. (2020). SEIPS 3.0: Human-centered design of the patient journey for patient safety. Applied Ergonomics, 84, 103033. doi.org/10.1016/j.apergo.2019.103033
12. Boztepe, H., Çınar Özbay, S., Akçam, A., & Kanbay, Y. (2023). The pediatric provider communication skills assessment scale. *Journal of Child and Adolescent Psychiatric Nursing, 36*(4), 307–315. doi.org/10.1111/jcap.12433
13. Makoul, G. (2001). Essential elements of communication in medical encounters: The Kalamazoo consensus statement. *Academic Medicine, 76*(4), 390–393. doi.org/10.1097/00001888-200104000-00021

Table S2. Sampling adequacy and factorability indices for the 10-item Parent Satisfaction with Pediatric Care scale (EFA subsample, n=221)

**Index**	**Value**
Kaiser–Meyer–Olkin (KMO) measure	0.94
Bartlett's test of sphericity – χ^2 (df=45)	2620
Bartlett's test – p	<0.001
Determinant of the correlation matrix	6.5×10^{-6}

Table S3. Eigenvalues and percentage of variance explained for the 10-item scale (principal axis factoring, EFA subsample, n=221)

**Factor**	**Eigenvalue (initial)**	**% of variance**	**Cumulative %**
1	7.99	78.0	78.0
2	0.57	5.6	83.6
3	0.48	4.7	88.3
4	0.29	2.8	91.1
5	0.22	2.2	93.3
6	0.19	1.9	95.2
7	0.19	1.9	97.1
8	0.14	1.3	98.4
9	0.11	1.1	99.5
10	0.08	0.5	100.0

Table S4. Factor loadings and extracted communalities from EFA (one-factor solution, EFA subsample, n=221)

**Item**	**Brief content (abbreviated)**	**Factor loading (λ)**	**Communality (h^2, extracted)**
ITEM1	Information about child's illness	0.90	0.81
ITEM 2	Information about treatment and medications	0.91	0.82
ITEM 3	Doctors' ability to establish contact with child	0.79	0.63
ITEM 4	Involvement of parent in decisions	0.94	0.89
ITEM 5	Explanations of options for tests/treatment/prevention	0.99	0.99
ITEM 6	Respect shown by staff	0.80	0.64
ITEM 7	Response to expressed needs and requests	0.87	0.76
ITEM 8	Time devoted by staff	0.88	0.78
ITEM 9	Cooperation between providers / facilities	0.87	0.76
ITEM 10	Overall quality of care received by the child	0.81	0.66

Table S5. Global fit indices for one-factor CFA models (CFA subsample, n=341)

**Estimator**	**Model**	
	Ordinal CFA (DWLS/WLSMV, primary)	Continuous CFA (ML/MLR, sensitivity)
χ^2	41.82 (30)	75.94 (30)
p	0.074	<0.001
CFI	1.000	0.987
TLI	1.000	0.980
RMSEA (90% CI)	0.034 (0 – 0.057)	0.067 (0.048–0.086)
p(close fit, RMSEA≤0.05)	0.860	0.070
SRMR	0.020	0.020
Robust CFI	0.979	0.993
Robust TLI	0.968	0.989
Robust RMSEA (90% CI)	0.102 (0.074–0.130)	0.049 (0.008–0.078)

Table S6. Standardized factor loadings and R^2 for ZAD1–ZAD10 in ordinal and continuous CFA (CFA subsample, n=341)

**Item**	**Ordinal CFA λ (Std.all)**	**Ordinal R^2**	**Continuous CFA λ (Std.all)**	**Continuous R^2**
ITEM 1	0.889	0.79	0.840	0.71
ITEM2	0.877	0.77	0.823	0.68
ITEM 3	0.913	0.83	0.857	0.74
ITEM 4	0.921	0.85	0.869	0.76
ITEM 5	0.904	0.82	0.867	0.75
ITEM 6	0.908	0.82	0.841	0.71
ITEM7	0.885	0.78	0.837	0.70
ITEM 8	0.903	0.82	0.854	0.73
ITEM 9	0.833	0.70	0.765	0.59
ITEM 10	0.867	0.75	0.799	0.64

Table S7. Correlated residuals specified in the CFA models (CFA subsample, n=341)

**Item pair (error covariance)**	**Ordinal model – standardized residual covariance**	**Continuous model – standardized residual covariance**	**Interpretation**
ITEM 1 ↔ ITEM2	0.72	0.55	Overlapping content about information on illness vs treatment/medications
ITEM4 ↔ ITEM5	0.54	0.35	Shared focus on involvement in decisions and explanation of options
ITEM 6 ↔ ITEM 7	0.50	0.38	Respect and responsiveness to needs/requests
ITEM7 ↔ ITEM 8	0.46	0.29	Responsiveness and time devoted
ITEM 9 ↔ ITEM10	0.31	0.25	Coordination across providers and overall quality of care

Table S8. Detailed hierarchical regression modeling of Parental Cumulative Satisfaction Pediatric Care Scale (PCS-PC) score

Model	Variable	Categories of variables	B (SE)	Beta	95%CI	p
Model 1	Age of parents		0.01 (0.01)	0.04	(-0.01 - 0.02)	0.445
	Sex	male#				
		female	-0.02 (0.10)	-0.01	(-0.23 - 0.18)	0.816
	Marital status	other#				
		married or in partnership	-0.27 (0.13)	-0.12	(-0.52 - -0.03)	0.031
		single	-0.35 (0.22)	-0.09	(-0.79 - 0.09)	0.120
	Place of residence	rural#				
		urban <10,000	0.07 (0.16)	0.02	(-0.25 - 0.39)	0.655
		urban 10,000–100,000	-0.14 (0.11)	-0.07	(-0.35 - 0.07)	0.195
		urban 100,000–500,000	-0.15 (0.12)	-0.07	(-0.38 - 0.09)	0.216
		urban >500.000	-0.22 (0.11)	-0.11	(-0.43 - 0.000)	0.050
	Education	university Master's#				
		lower than secondary	0.35 (0.12)	0.15	(0.10 - 0.59)	0.005
		secondary	0.17 (0.10)	0.09	(-0.03 - 0.37)	0.100
		post-secondary non-university	0.23 (0.14)	0.08	(-0.05 - 0.51)	0.110
		university Bachelor's	0.46 (0.16)	0.15	(0.15 - 0.78)	0.004
	Income	2001–3000 PLN#				
		≤1500 PLN	-0,41 (0.13)	-0.20	(-0.66 - -0.16)	0.001
		1501–2000 PLN	-0.16 (0.11)	-0.08	(-0.38 - 0.06)	0.164
		>3000 PLN	0.04 (0.12)	0.02	(-0.20 - 0.28)	0.739
		refusal to reveal	-0.07 (0.12)	-0.04	(-0.31 - 0.16)	0.546
	Vocational status	employee #				
		entrepreneur or farmer	0.14 (0.10)	0.07	(-0.06 - 0.35)	0.166
		unemployed or part-time job	0.08 (0.14)	0.03	(-0.20 - 0.36)	0.588
		other	-0.09 (0.09)	-0.04	(-0.28 - 0.11)	0.386
Model 2	Age of parents		0.002 (0.01)	0.02	(-0.01 - 0.02)	0.779
	Sex	male#				
		female	-0.02 (0.10)	-0.01	(-0.23 - 0.02)	0.853
	Marital status	other#				
		married or in partnership	-0.23 (0.13)	-0.10	(-0.48 - 0.19)	0.072
		single	-0.28 (0.23)	-0.07	(-0.73 - 0.17)	0.217
	Place of residence	rural#				
		urban <10,000	0.07 (0.16)	0.02	(-0.25 - 0.39)	0.681
		urban 1,000–100,000	-0.12 (0.11)	-0.06	(-0.33 - 0.09)	0.274
		urban 100,000–500,000	-0.10 (0.12)	-0.04	(-0.34 - 0.14)	0.413
		urban >500,000	-0.21 (0.11)	-0.10	(-0.43 - 0.01)	0.063
	Education	university Master's#				
		lower than secondary	0.35 (0.13)	0.16	(0.11 - 0.60)	0.005
		secondary	0.14 (0.10)	0.08	(-0.06 - 0.35)	0.169
		post-secondary non-university	0.20 (0.15)	0.07	(-0.09 - 0.48)	0.177
		university Bachelor's	0.44 (0.16)	0.14	(0.12 - 0.77)	0.007
	Income	2001–3000 PLN#				

		≤1500 PLN	-0.39 (0.13)	-0.19	(-0.64 - -0.14)	0.002
		1501–2000 PLN	-0.14 (0.11)	-0.07	(-0.37 - 0.08)	0.206
		>3000 PLN	0.05 (0.13)	0.02	(-0.20 - 0.30)	0.701
		refusal to reveal	-0.07 (0.12)	-0.03	(-0.30 - 0.17)	0.585
	Vocational status	employee #				
		entrepreneur or farmer	0.14 (0.10)	0.07	(-0.06 - 0.35)	0.174
		unemployed or part-time job	0.12 (0.15)	0.04	(-0.17 - 0.40)	0.421
		other	-0.06 (0.10)	-0.03	(-0.25 - 0.14)	0.575
	Age of a child		0.01 (0.01)	0.04	(-0.02 - 0.03)	0.545
	Duration of disease		-0.01 (0.01)	-0.05	(-0.03 - 0.01)	0.395
	Emergency	no use#				
		1	-0.08 (0.11)	-0.04	(-0.30 - 0.14)	0.461
		>1	-0.03 (0.11)	-0.02	(-0.25 - 0.19)	0.772
	Hospitalization	no#				
		1	-0.01 (0.11)	-0.01	(-0.23 - 0.20)	0.908
		>1	-0.13 (0.11)	-0.07	(-0.35 - 0.09)	0.258
	IBD	no#				
		yes	0.10 (0.10)	0.06	(-0.10 - 0.30)	0.314
Model 3	Age of parents		0.001 (0.01)	0.01	(-0.01 - 0.02)	0.871
	Sex	male#				
		female	0.06 (0.10)	0.03	(-0.15 - 0.26)	0.584
	Marital status	other#				
		married or in partnership	-0.29 (0.13)	-0.13	(-0.54 - -0.04)	0.021
		single	-0.26 (0.22)	-0.07	(-0.70 - 0.17)	0.236
	Place of residence	rural#				
		urban <10.000	0.07 (0.16)	0.02	(-0.24 - 0.38)	0.670
		urban 10.000–100.000	-0.10 (0.10)	-0.05	(-0.31 - 0.10)	0.330
		urban 100.000–500.000	-0.08 (0.12)	-0.04	(-0.32 - 0.15)	0.458
		urban >500.000	-0.24 (0.11)	-0.12	(-0.45 - -0.03)	0.029
	Education	university Master’s#				
		lower than secondary	0.30 (0.12)	0.13	(0.06 - 0.54)	0.015
		secondary	0.13 (0.10)	0.07	(-0.07 - 0.33)	0.193
		post-secondary non-university	0.14 (0.14)	0.05	(-0.14 - 0.42)	0.321
		university Bachelor’s	0.41 (0.16)	0.13	(0.10 - 0.72)	0.010
	Income	2001–3000 PLN#				
		≤1500 PLN	-0.34(0.13)	-0.16	(-0.59 - -0.10)	0.007
		1501–2000 PLN	-0.13 (0.11)	-0.07	(-0.35 - 0.08)	0.226
		>3000 PLN	0.02 (0.12)	0.01	(-0.23 - 0.26)	0.900
		refusal to reveal	-0.06 (0.12)	-0.03	(-0.29 - 0.17)	0.623
	Vocational status	employee #				
		entrepreneur or farmer	0.09 (0.10)	0.05	(-0.11 - 0.29)	0.368
		unemployed or part-time job	0.15 (0.14)	0.06	(-0.13 - 0.43)	0.289
		other	-0.08 (0.10)	-0.04	(-0.28 - 0.11)	0.392
	Age of a child		0.004 (0.01)	0.02	(-0.02 - 0.03)	0.727
	Duration of disease		-0.01 (0.01)	-0.05	(-0.03 - 0.01)	0.400

	Emergency	no use#				
		1	-0.07 (-0.11)	-0.03	(-0.28 - 0.15)	0.541
		>1	-0.04 (0.11)	-0.02	(-0.26 - 0.18)	0.720
	Hospitalization	no#				
		1	0.03(0.11)	0.01	(-0.18 - 0.23)	0.810
		>1	-0.06 (0.11)	-0.03	(-0.28 - 0.16)	0.577
	IBD	no#				
		yes	0.07(0.10)	0.04	(0.13 - 0.16)	
	PSS-10 score		-0.03 (0.01)	-0.24	(-0.05 - -0.02)	0.000
Model 4	Age of parents		-0.001 (0.01)	-0.01	(-0.02 - 0.02)	0.917
	Sex	male#				
		female	0.03 (0.10)	0.02	(-0.17 - 0.24)	0.750
	Marital status	other#				
		married or in partnership	-0.34 (0.13)	-0.15	(-0.59 - -0.10)	0.007
		single	-0.32 (0.22)	-0.08	(-0.75 - 0.12)	0.150
	Place of residence	rural#				
		urban <10,000	0.05 (0.16)	0.02	(-0.26 - 0.36)	0.732
		urban 10,000–100,000	-0.10 (0.11)	-0.05	(-0.30 - 0.11)	0.361
		urban 100,000–500,000	-0.11 (0.12)	-0.05	(-0.34 - 0.12)	0.364
		urban >500,000	-0.25 (0.11)	-0.12	(-0.47 - 0.04)	0.022
	Education	university Master's#				
		lower than secondary	0.25 (0.13)	0.11	(-0.003 - 0.50)	0.053
		secondary	0.10 (0.10)	0.05	(-0.10 - 0.30)	0.338
		post-secondary non-university	0.11 (0.14)	0.04	(-0.17 - 0.39)	0.437
		university Bachelor's	0.40 (0.16)	0.12	(0.09 - 0.71)	0.013
	Income	2001–3000 PLN#				
		≤1500 PLN	-0.37 (0.13)	-0.18	(-0.61 - -0.12)	0.003
		1501–2000 PLN	-0.15 (0.11)	-0.08	(-0.37 - 0.07)	0.190
		>3000 PLN	0.003 (0.12)	0.001	(-0.24 - 0.25)	0.983
		refusal to reveal	-0.09 (0.12)	-0.04	(-0.32 - 0.14)	0.447
	Vocational status	employee #				
		entrepreneur or farmer	0.13 (0.10)	0.06	(-0.08 - 0.33)	0.219
		unemployed or part-time job	0.16 (0.14)	0.06	(-0.11 - 0.44)	0.245
		other	-0.10 (0.10)	-0.05	(-0.29 - 0.09)	0.293
	Age of a child		0.004 (0.01)	0.02	(-0.02 - 0.03)	0.741
	Duration of disease		-0.01 (0.01)	-0.04	(-0.03 - 0.02)	0.483
	Emergency	no use#				
		1	-0.04 (0.11)	-0.02	(-0.25 - 0.18)	0.728
		>1	-0.01 (0.11)	-0.004	(-0.23 - 0.21)	0.941
	Hospitalization	no#				
		1	0.04 (0.11)	0.02	(-0.07 - 0.25)	0.714
		>1	-0.04 (0.11)	-0.02	(-0.26 - 0.18)	0.705
	IBD	no#				
		yes	0.05 (0.01)	0.03	(-0.14 - 0.25)	0.583
	PSS-10 score		-0.03 (0.01)	-0.22	(-0.04 - -0.02)	0.000
	Daily Internet use	>90 min#				
		≤30 min	0.12 (0.13)	0.06	(-0.14 - 0.38)	0.363

		31–45 min	0.01 (0.13)	0.01	(-0.25 - 0.27)	0.925
		46–60 min	-0.17 (0.12)	-0.09	(-0.40 - 0.06)	0.155
		61–90 min	0.02 (0.13)	0.01	(-0.23 - 0.27)	0.859
	Daily social media use	15–30 min #				
		no use	0.05 (0.12)	0.02	(-0.19 - 0.30)	0.668
		<15 min	0.06(0.11)	0.03	(-0.15 - 0.27)	0.582
		31–60 min	0.07 (0.10)	0.04	(-0.14 - 0.27)	0.520
		>60 min	0.23 (0.19)	0.07	(-0.14 - 0.60)	0.224
	eHL		-0.08(0.06)	-0.06	(-0.20 - 0.05)	0,241
	HL	sufficient #				
		inadequate	-0.64 (0.17)	-0.18	(-0.98 - 0.31)	0.000
		problematic	-0.08 (0.10)	-0.04	(-0.28 - 0.13)	0.451
		undetermined	-0.15 (0.12)	-0.06	(-0.38 - 0.08)	0.205

Abbreviations: PSS - perceived stress scale, eHL - e-health literacy, HL - health literacy, # - reference category
